# Quantification of the Regional Properties of Gastric Motility Using Dynamic Magnetic Resonance Images

**DOI:** 10.1109/OJEMB.2023.3261224

**Published:** 2023-03-23

**Authors:** Saeed Hosseini, Recep Avci, Niranchan Paskaranandavadivel, Vinod Suresh, Leo K. Cheng

**Affiliations:** Auckland Bioengineering InstituteUniversity of Auckland 1415 Auckland 1010 New Zealand; Riddet Institute 540798 Palmerston North 4474 New Zealand; Auckland Bioengineering InstituteUniversity of Auckland 1415 Auckland 1010 New Zealand; Auckland Bioengineering InstituteUniversity of Auckland 1415 Auckland 1010 New Zealand; Department of Engineering ScienceUniversity of Auckland 1415 Auckland 1010 New Zealand; Auckland Bioengineering InstituteUniversity of Auckland 1415 Auckland 1010 New Zealand; Riddet InstitutePalmerston North 93874 4474 New Zealand

**Keywords:** Contraction map, gastric motility index, magnetic resonance imaging, motion correction

## Abstract

*Goal*: To quantify the regional properties of gastric motility from free-breathing dynamic MRI data. *Methods*: Free-breathing MRI scans were performed on 10 healthy human subjects. Motion correction was applied to reduce the respiratory effect. A stomach centerline was automatically generated and used as a reference axis. Contractions were quantified and visualized as spatio-temporal contraction maps. Gastric motility properties were reported separately for the lesser and greater curvatures in the proximal and distal regions of the stomach. *Results*: Motility properties varied in different regions of the stomach. The mean contraction frequencies for the lesser and greater curvatures were both 3.1±0.4 cycles per minute. The contraction speed was significantly higher on the greater curvature than the lesser curvature (3.5±0.7 vs 2.5±0.4 mm/s, p<0.001) while contraction size on both curvatures was comparable (4.9±1.2 vs 5.7±2.4 mm, p = 0.326). The mean gastric motility index was significantly higher in the distal greater curvature (28.13±18.89 mm^2^/s) compared to the other regions of the stomach (11.16–14.12 mm^2^/s). *Conclusions*: The results showed the effectiveness of the proposed method for visualization and quantification of motility patterns from MRI data.

## I. Introduction

The mixing and grinding of food particles inside the stomach are carried out by muscular wave-like contractions coordinated by a combination of rhythmic electrical slow waves events, neural activity, and hormones [1]. In the healthy stomach, the contractions originate in the upper corpus and propagate in rings toward the pylorus, exerting forces to mix and disintegrate the digested food [2]. In addition, the breakdown of orally administered drugs is highly affected by gastric motility patterns [3].

Measurements of gastric motor activity can be used to diagnose and understand gastric motility disorders. A number of methods and techniques have been proposed in the assessment of gastric motility disorders such as gastroparesis and functional dyspepsia [4]. Gastroparesis is defined by delayed gastric emptying (greater than 10% retention after 4 hours [5]) and associated with debilitating symptoms such as nausea, vomiting and bloating. Scintigraphy is currently the gold standard for the measurement of gastric emptying rates. However, a lack of standardization obscures the clinical utility of this test [6]. In addition, it is a time-consuming process, and exposes the patient to radiation [7]. In addition, it has been shown that some of these gastric disorders could easily be confused with each other, and gastric emptying alone is insufficient to distinguish between these diseases [8]. Intraluminal pressure recording using a barostat is another method to measure the mechanical activity of the stomach. However, the barostat balloon can interfere with gastric accommodation and motility [9].

Magnetic resonance imaging (MRI) is an alternative technique that shows promise for quantifying emptying and gastric motility properties such as frequency and propagation speed [10], [11], [12], [13], [14], [15]. However, analyzing the data from MRI remains challenging [16]. Previously, gastric motor activity has been measured using two manually-drawn lines for the stomach axis as references. The distance from the stomach wall to these reference lines was used to calculate the frequency and speed of the contractions [10]. In a later work, a manually-drawn line was used as the antrum axis, and thresholds were applied for calculations of gastric motility [17]. In addition, tagging lines used in cardiac MRI to determine the speed and intensity of contractions [18] were employed to derive motility metrics [19], [20].

Free-breathing MRI scans of gastric motility were also used to quantify contraction speed using an axis drawn through the center of the antrum [21]. Other studies have used manually prescribed lines as the reference for the propagation speed calculations of breath-hold images [12], [22], [23], [24]. More recently, researchers have investigated the use of an automated method for analyzing dynamic 3-dimensional MRI data [25], [26], [27]. Although 3-dimensional imaging has the significant advantage of imaging the entire stomach, in comparison to 2-dimensional imaging the temporal resolution is significantly lower and the computational cost of analyzing this data is much higher.

Despite technological advances, a number of key steps in the analysis pipeline are still performed manually, hindering efficiency. In this study, a stomach centerline was automatically estimated and regional variations in the contractions on the lesser and greater curvatures were quantified using free-breathing MRI data.

## II. Materials and Methods

### A. Data Acquisition

Ethical approval was provided by the University of Auckland Human Participants Ethics Committee, and all participants provided informed consent. Ten participants were selected from healthy and non-pregnant volunteers between the ages of 18–65 (5 male, 5 female, 28±4 years).

All participants fasted for at least 6 hours prior to MRI. Fifteen minutes before the scans, the participants consumed 500 ml pineapple juice and a solid meal i.e., New Zealand style pie (approximately 375 g and 750 kcals). Pineapple juice is a natural contrast agent and used as an alternative to gadolinium [28].

The participants laid supine in the MRI machine. The orientation of the imaging slices was selected by exploring an imaging angle that provided good coverage of the distal stomach. First, images in the transverse plane were captured to locate the distal antrum and pylorus. Then, a semi-coronal plane cutting the antrum in half was selected to capture the pylorus, distal antrum and both lesser and greater curvatures of the stomach, while maximizing the overall view of the stomach. A 17 s breath-hold session was first performed, and stomach border clarity and contractile activity on the greater and lesser curvatures (i.e., whether they terminate at the distal stomach at the same time) were examined to confirm the plane selection. Finally, a series of 100 MR images was captured during an approximately 60 s of free-breathing session. During the free-breathing sessions, participants were instructed to take shallow and consistent breaths. This approach minimized the movement of the stomach in and out of the semi-coronal imaging plane.

MRI scans were performed using either 1.5 Tesla Avanto or 3 Tesla Skyra machines (Siemens, Erlangen, Germany) based on availability and a gradient echo sequence of fast imaging with steady-state free precession – TrueFISP was used [29]. The imaging frequency for the 1.5 Tesla machine was 92 images per minute (1.5 Hz), while the echo time (TE) was 1.35 ms and repetition time (TR) was 500 ms. For the 3 Tesla machine, the imaging frequency was 109 images per minute (1.8 Hz) with the TE of 1.78 ms and TR of 369 ms. The flip angle was 60° in both machines and a slice thickness of 4 mm was used. Image resolutions were 416 × 416 and 256 × 204 for the 1.5 Tesla and the 3 Tesla systems with the pixel size of 0.96 mm and 1.47 mm, respectively. No significant differences in image quality were observed between the data obtained from the 2 MRI scanners.

### B. Motion Correction

To remove breathing artifacts from the image sequences, 4 landmark points (3 on the liver and 1 point on the gall bladder) that were visible throughout the whole sequence were manually selected on each image. The landmark points on the first image in each series were used as the reference. An optimization procedure using the *fminsearch* function in MATLAB R2018a (MathWorks, Natick, MA, USA) was employed to determine the number of pixels to translate (Δx, Δy) and the angle (θ) to rotate the image:

\begin{align*}
& {\rm{\Delta x}},{\rm{\Delta y}},\ \theta = \\
& \mathop {\rm argmin}\limits_{x^{\prime},{\rm{y^{\prime}}},{\rm{\theta ^{\prime}}}} \sum\limits_{i = 1}^4 {{{ \left({{P}_{t,i}\left({x + x^{\prime},y + y^{\prime}} \right)R\left({\theta ^{\prime}} \right) - {P}_{1,i}\left({x,y} \right)} \right)}}^2} \tag{1}
\end{align*}

where *i* corresponds to each reference point, *P*_1_*_,i_* and *P_t,i_* are the *i-th* reference point on the first image and the target image at time *t*, respectively, while *R* is the rotation matrix with respect to the origin. Once the optimal correction parameters (Δx, Δy, θ) were identified, they were applied to the target images to minimize movement between each frame.

The effectiveness of the motion correction algorithm to remove breathing artifact was quantified by comparing the mean temporal derivative of root mean square variance (DVARS) [30] over pixels along a vertical line that crosses the stomach in the MR images computed as follows:

\begin{equation*}
\text{DVARS} = \frac{1}{{\mathrm{T} - 1}}\sum\limits_{t = 2}^T {\left({\sqrt {\frac{1}{n}\sum\limits_{p = 1}^n {{{\left({{I}_{\mathrm{p},\mathrm{t}} - {I}_{\mathrm{p},\mathrm{t} - 1}} \right)}}^2} } } \right)} \tag{2}
\end{equation*}

where *t* is the image number in the time series, *T* is the total number of images, *n* is the number of the pixels in the vertical line, *p* is the pixel number, and *I* is the pixel intensity.

### C. Centerline Estimation

**Fig. 1.**
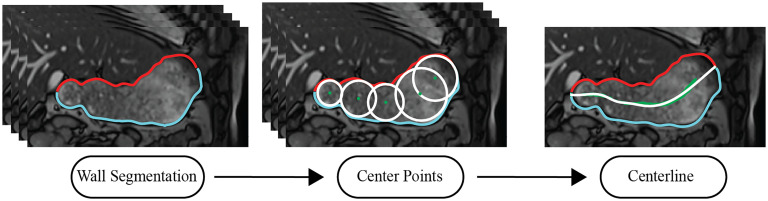
Steps used to generate the centerline of the stomach. First, the lesser and greater curvatures of the stomach were segmented and circles that fit within the stomach were determined for each time-step. Then, a line was fitted to the centers of all the circles.

A stomach centerline corresponding to the longitudinal axis of the stomach was computed to act as a reference axis to determine the magnitude of contractions. First, the lesser and greater curvatures of the stomach were segmented (see Fig. 1). Then, cubic splines were fitted to each curvature and used to create 200 equally spaced points, termed wall-points. The corresponding wall-points on both curvatures were paired, and the distance between each pair was calculated, along with a point located midway between the two curvatures (termed middle-points). Next, circles centered at the middle-point with a diameter of 90% of the distance between the two corresponding wall-points were generated. If the entire circle was located within the stomach outline, the middle-point was classified as a valid middle-point, otherwise it was discarded. Finally, the most distal and proximal points on the lesser and greater curvatures were included as valid middle-points. The process was repeated for all time steps.

Once the valid middle-points were identified for all the MR images in the time series, a centerline of the stomach was estimated by fitting a 5^th^ order polynomial to all valid middle-points (Fig. 1). The centerline was then used to generate 200 equally spaced middle-points.

### D. Contraction Quantification and Visualization

The centerline was used as a fixed reference axis for the wall motility calculations. First, a reference stomach radius for each wall-point was computed across all time steps using (3).

\begin{equation*}
Reference\ Radius\left(n \right) = \sum\limits_{t = 1}^T {\frac{{\text{Dc}\left({n,t} \right)}}{T}} \tag{3}
\end{equation*}

where *n* shows the point number in the lesser or greater curvature, *Dc(n,t)* is the distance of the *n-th* wall-point to the corresponding center-point. Then, the contraction size for each wall-point at time *t* was calculated as follows:

\begin{equation*}
\begin{array}{l} Contraction\ Size\left({n,t} \right) = \\ Reference\ Radius\left(n \right) - Dc\left({n,t} \right) \end{array} \tag{4}
\end{equation*}

Subsequently, the percentage occlusion of the contraction was computed using (5).

\begin{equation*}
\begin{array}{l} Percentage\ Occlusion\left({n,t} \right) = \\ \frac{{Contraction\ Size\left({n,t} \right)}}{{Reference\ Radius\left(n \right)}}\ \times 100 \end{array} \tag{5}
\end{equation*}

**Fig. 2.**
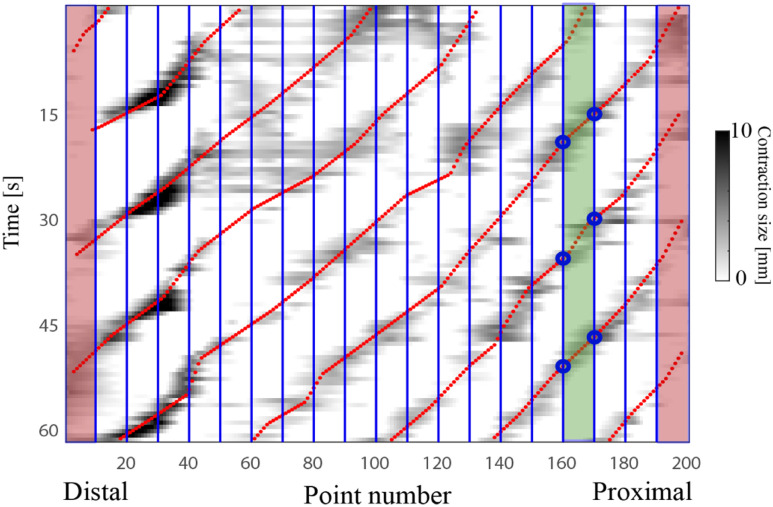
Representative contraction map from one subject. The map was spatially divided into 20 regions (blue lines), and the first and last regions were excluded from further analysis (shaded red). Each contraction wave (dark shading) was tracked (red points) to quantify contraction speed.

The contraction size and percentage occlusion were calculated for all time-steps and were displayed in the form of a contraction map to visualize and quantify contraction speeds and directions (see Fig. 2). The contraction map where the greyscale field indicates magnitude of contraction (dark grey indicates strongest contractions) shows the variability of contractions in different regions of the stomach and over time.

### E. Quantification of Gastric Motility

The contraction maps were used to quantify the location and propagation speed of contraction waves. First, the contraction waves were identified and tracked by manually marking 10 points along the wave based upon contraction intensity and then interpolating them (red line in Fig. 2). The map was then spatially divided into 20 regions (blue vertical lines in Fig. 2). The first and last regions were discarded to avoid boundary effects related to the high curvatures at the distal and proximal stomach (shaded red in Fig. 2). The wave speed in each region was calculated as follows:

\begin{equation*}
Contraction\ Speed = \frac{{region\_width}}{{t2 - t1}} \tag{6}
\end{equation*}

where *region_width* is the distance that the wave travelled from time *t1* to *t2* computed using the mean length of the lesser or greater curvature for all time steps.

The mean wave speed was computed for the regions where multiple contraction waves were present. The frequency of the waves was measured automatically by calculating the average time between each two consecutive waves within a region (i.e., the vertical distance between each red line). In addition, the gastric motility index (GMI) [19] was calculated for each participant on the lesser and greater curvatures in the proximal and distal regions of the stomach as follows:

\begin{equation*}
GMI = \overline {Contraction\ Speed} \times \overline {Contraction\ Size} \tag{7}
\end{equation*}

where $\overline {Contraction\ Speed} $ and $\overline {Contraction\ Size} $ are the mean speed and size in the region of interest.

### F. Statistical Analysis

Data across subjects are summarized as mean ± standard deviation. Statistical tests were performed using MATLAB R2018a with a significance level (alpha) of 0.05. Shapiro-Wilk test was first employed to examine the normality of data. To quantify the differences between free-breathing, motion removed, and breath-hold MR images, the DVARS values were compared using the Kruskal-Wallis test followed by a post-hoc test. Contraction metrics were analyzed along 18 stomach regions for both lesser and greater curvatures of 10 subjects. Trends from distal to proximal stomach were investigated by performing a polynomial fitting to the mean data of all regions and computing the Spearman correlation. The differences in contraction metrics (i.e., contraction speed, contraction size, and occlusion) between the lesser and greater curvatures were investigated using a two-sample t-test. GMI metrics computed for the proximal and distal regions of the lesser and greater curvatures were compared using Analysis of Variance (ANOVA) followed by a post-hoc test.

## III. Results

All 10 subjects participating the study tolerated the MRI study, and the scans were completed without any complications. Clear stomach borders were observed, and contractions were present in all cases.

### A. Motion Correction

**Fig. 3.**
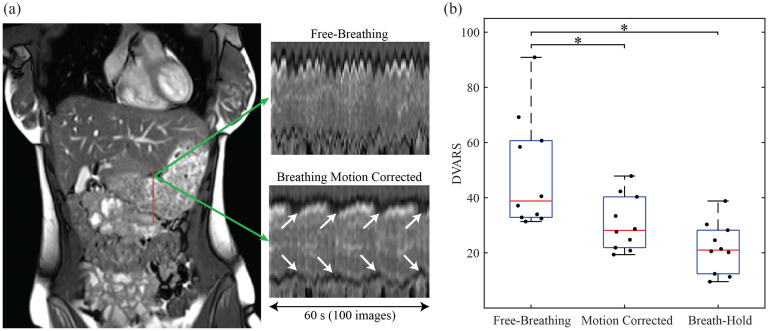
Motion correction of breathing artifacts. (a) A coronal MR image, along with the change in pixel intensity for a vertical line segment (red line) before and after motion correction. The contractions (white arrows) were more evident after motion correction. (b) Comparison of the DVARS metric before and after motion correction, and with corresponding breath-hold data from each participant. ^*^ indicates p<0.05 from a post-hoc analysis following the Kruskal-Wallis test (N=10).

Motion correction was performed for all datasets and resulted in improved consistency of stomach position. The effectiveness of the motion correction algorithm is illustrated in Fig. 3. The variation in pixel intensity of vertical line of pixels (red line) before and after motion correction is shown in Fig. 3(a). The raw data shows large cyclic movements that obscure the contractions of the stomach, whereas the contractions (white arrows) are more evident in the motion corrected image. The DVARS metric was used to quantify reduction in motion for all subjects as shown in Fig. 3(b). For comparison, the DVARS metric was also calculated for a series of 20 breath-hold images of each participant in the same slice position. The mean DVARS value for the motion removed images was significantly lower than free-breathing images (30.7±9.9 vs 48.7±20.2, p < 0.001) and was comparable to the breath-hold images (21.7±9.2, p = 0.224).

### B. Contraction Analysis

**Fig. 4.**
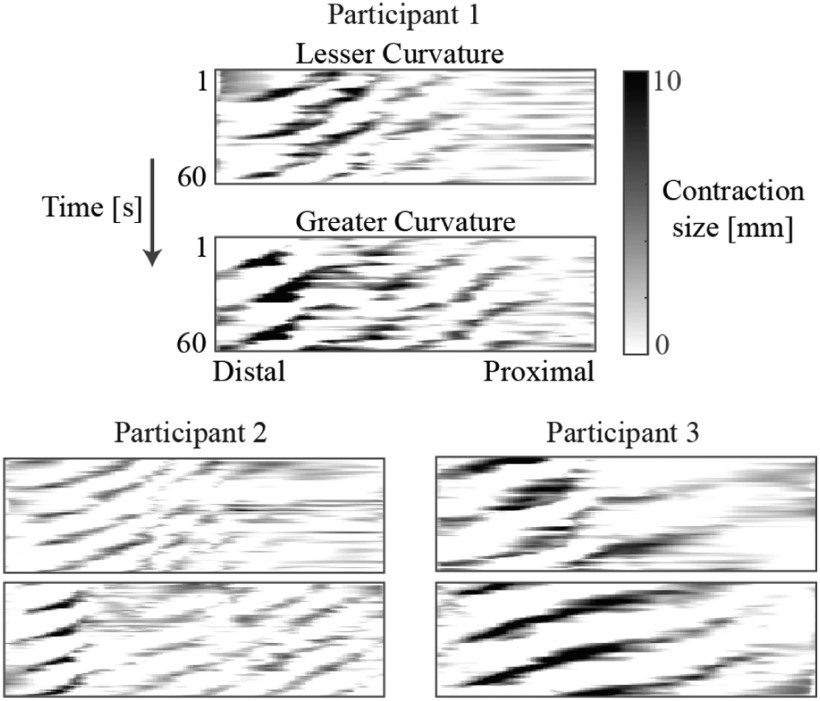
Contraction maps corresponding to the lesser curvature (upper map) and greater curvature (lower map) for 3 representative participants. The normalized length of the curvature is shown on the horizontal axis and time is given on the vertical axis.

Representative contraction maps for 3 participants are shown in Fig. 4, where clear contraction patterns from the proximal stomach to the distal stomach can be seen. Out of these 3 participants, Participant 3 had the largest contraction size (darker regions in the maps). The average contraction speed was the highest for Participant 1 (the slope of the contraction waves).

For all subjects studied, the mean lengths of the lesser and greater curvatures were 200±22 mm and 286±34 mm, respectively, while the estimated centerlines had a length of 197±20 mm. The contraction frequencies for the lesser and greater curvatures were both 3.1±0.4 cycles per minute. The GMI values varied across the stomach where the distal stomach on the greater curvature had the highest GMI (28.13±18.89 mm^2^/s), which was significantly higher than the GMI in the proximal stomach on the greater curvature (14.12±6.01 mm^2^/s, p = 0.023) and on both the proximal (11.16±2.98 mm^2^/s, p = 0.004) and distal (12.61±5.36 mm^2^/s, p = 0.010) regions of the lesser curvature. The overall GMI calculated in this study was 16.2±7.1 mm^2^/s and was not significantly different from any of the local GMIs.

**Fig. 5.**
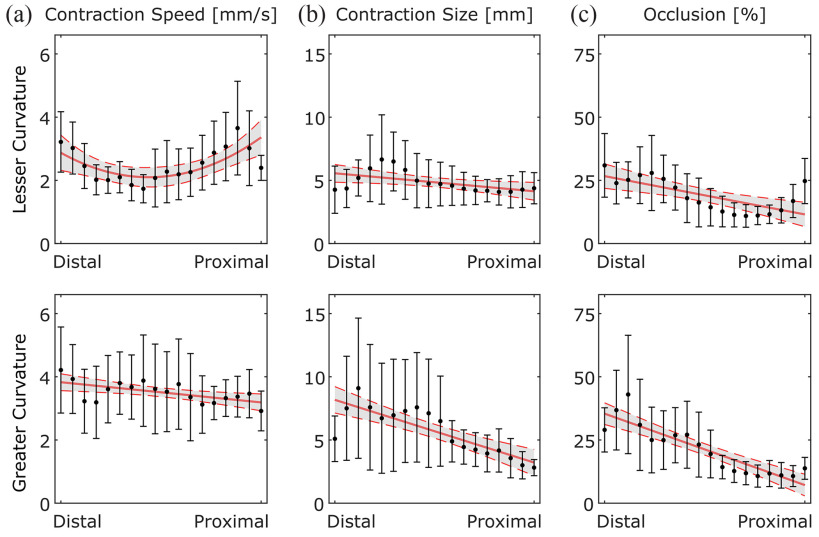
For different regions of the stomach along the lesser and greater curvatures, variation in (a) contraction speed, (b) contraction size, and (c) occlusion percentage are shown. The data points correspond to the mean value in each region while the error bars represent 1 standard deviation from the mean. The red lines represent the fitted model while shaded regions correspond to 95% confidence interval.

Fig. 5 shows the distribution and trends of contraction metrics along the stomach for all subjects. On the lesser curvature, the proximal and distal stomach had higher speeds compared to the mid-stomach. On the other hand, the speed on the greater curvature decreased from distal to proximal stomach (slope: **–** 0.038, p = 0.008) and a correlation coefficient (r) of **–** 0.567 (p = 0.016) as seen in Fig. 5(a). The greater curvature speeds varied less through the length of the curvature and were generally higher than lesser curvature wave speeds (3.5±0.7 mm/s vs 2.5±0.4 mm/s, p < 0.001).

The contraction size decreased from distal to proximal stomach for both the lesser curvature (slope = **–** 0.084, p = 0.022 and r = **–** 0.577, p = 0.014) and greater curvature (slope = –0.292, p < 0.001 and r = –0.851, p < 0.001) as seen in Fig. 5(b). The average size of the contractions in lesser curvature was comparable with the greater curvature (4.9±1.2 vs 5.7±2.4 mm, p = 0.326). The contraction size normalized by the average distance of the wall-point (occlusion) for lesser and greater curvatures are shown in Fig. 5(c). The mean occlusion percentages for lesser and greater curvatures were 19±5% and 21±7%, respectively (p = 0.452). Occlusion decreased from distal to proximal stomach in both the lesser curvature (slope = **–** 0.892, p = 0.001 and r = **–** 0.676, p = 0.003) and the greater curvature (slope = **–** 1.662, p < 0.001 and r = **–** 0.917, p < 0.001).

## IV. Discussion

In this study, a framework to analyze and quantify MR images of gastric motility was developed. Dynamic gastric MRIs were obtained from healthy volunteers. A motion correction algorithm was employed to reduce artifacts resulting from breathing. Gastric motility patterns were visualized using motility maps, and regional variations in contraction speed, contraction size and occlusion percentage were determined.

MR images had sufficient border clarity for manual segmentation without using a gadolinium-based contrast agent. Although our method segmented the stomach border manually, automated segmentation methods would help to reduce variability and bias. The motion correction algorithm was effective at reducing motion artifacts enabling free-breathing datasets with longer duration to be analyzed.

In this study, our primary focus was to select an imaging slice capturing the corpus and antrum where most contractions occur. The use of 3-dimensional MR imaging methods can provide a greater field of view and remove the need to carefully select an appropriate imaging plane [25], [26]. Our data was acquired every 0.65 s, whereas 3-dimensional methods have reported obtaining volumetric data every 4.2-7 s. Although gastric dynamics are relatively slow, contraction speeds of 3 mm/s indicate that blurring or distortions in the data may result with such low imaging frequencies. It remains uncertain if obtaining a larger field of view via volumetric imaging methods outweighs any reduction in imaging quality due to resolution and/or imaging time.

In this study, we showed the capability of a semi-automatic method to quantify the contractions of the stomach from MRI data and determine local contraction properties on the lesser and greater curvature. Our method increased the efficiency of motility calculations as the centerline was automatically calculated based on the anatomical shape of each stomach, whereas previous studies used manually drawn centerlines [22], [23] or straight lines [2], [31], which would not accurately represent the stomach curvature. Using a straight line as the reference of the measurement can lead to inaccuracies, whereas manual placement of centerlines is prone to human errors. The novel method developed in this study reduces the need for manual operations and subjective decisions and provides greater benefits in clinical usage.

Separating the lesser and greater curvature enabled individual contractions in the contraction maps to be distinguished and contraction properties to be quantified. Previous studies have combined the contractions of the lesser and greater curvature seen in the MRI slice (and therefore analyzed the variation in the diameter of the stomach) [21], [23], [24], [32]. However, as the contraction points may not always align well, increased error can result. Analyzing each curvature individually removes the matching errors and allows variations in the contractions in the lesser and greater curvature to be quantified [23].

The results showed the overall occlusion of the contractions in the pylorus area is higher which was previously believed to be merely due to narrowing of the stomach near the pylorus [21]. However, our findings suggest that it is also affected by the size of the contractions increasing near the distal side of the stomach. The number of simultaneous waves, mean frequency of the contractions, average speeds, size, and occlusion reported in this study align with previously reported numbers from MRI studies of gastric motility [12], [25], [33]. High-resolution mapping of the electrical activity in the stomach has shown an increased speed and amplitude of the slow wave in the distal antrum compared to the proximal antrum or corpus [34]. Our results also showed increasing contraction speeds and sizes in the distal antrum, but the increase was more gradual.

In addition, we showed that the speed and size of the contractions are not constant along the stomach and vary based on the points of measurements. This can affect quantification metrics such as the GMI [19], which assumed constant propagation speed throughout the stomach [21], [35]. The overall mean GMI calculated in this study was in accordance with previously reported values [36]. However, the GMI only incorporates a global averaged value for speed and contraction size for the entire stomach. As shown by the GMI values computed for separate regions of the stomach, a global GMI does not account for regional variations and likely underestimates the true motility characteristics of the stomach. Therefore, new motility metrics should consider taking into account the spatially varying properties identified in this study.

In the future, incorporating automated methods for segmentation of the stomach wall and for tracking the motility maps will help increase the efficiency and reliability of the proposed methods and makes it more suitable for clinical application. In addition, the contraction maps were normalized along the curvature length and the method can be improved by registering measurement points to the distal stomach. The participants in this study were selected from healthy volunteers and we have established the parameters corresponding to normal motility patterns. The proposed methods now await application to subjects with functional motility disorders.

## V. Conclusion

Dynamic MRI data were acquired from healthy subjects. Gastric motility patterns were extracted from the MRI data using semi-automatic methods. Methods were able to quantify motility patterns and show variation in different regions of the stomach. Overall, these methods advance MRI as a reliable technique for quantifying gastric motility patterns and provide an improved understanding of gastric motility patterns.
